# Mobile phone text messaging interventions for HIV and other chronic diseases: an overview of systematic reviews and framework for evidence transfer

**DOI:** 10.1186/s12913-014-0654-6

**Published:** 2015-01-22

**Authors:** Lawrence Mbuagbaw, Sara Mursleen, Lyubov Lytvyn, Marek Smieja, Lisa Dolovich, Lehana Thabane

**Affiliations:** Department of Clinical Epidemiology and Biostatistics, McMaster University, Hamilton, ON Canada; Biostatistics Unit, Father Sean O’Sullivan Research Centre, St Joseph’s Healthcare-Hamilton, Hamilton, ON Canada; Centre for Development of Best Practices in Health, Yaoundé Central Hospital, Yaoundé, Cameroon; St. Joseph’s Healthcare Hamilton, Hamilton, ON Canada; Department of Family Medicine, McMaster University, McMaster Innovation Park, Hamilton, ON Canada; Departments of Paediatrics and Anaesthesia, McMaster University, Hamilton, ON Canada; Centre for Evaluation of Medicine, St Joseph’s Healthcare-Hamilton, Hamilton, ON Canada; Population Health Research Institute, Hamilton Health Sciences, Hamilton, ON Canada

**Keywords:** Text message, HIV chronic disease, Evidence transfer, Overview

## Abstract

**Background:**

Strong international commitment and the widespread use of antiretroviral therapy have led to higher longevity for people living with human immune deficiency virus (HIV). Text messaging interventions have been shown to improve health outcomes in people living with HIV. The objectives of this overview were to: map the state of the evidence of text messaging interventions, identify knowledge gaps, and develop a framework for the transfer of evidence to other chronic diseases.

**Methods:**

We conducted a systematic review of systematic reviews on text messaging interventions to improve health or health related outcomes. We conducted a comprehensive search of PubMed, EMBASE (Exerpta Medica Database), CINAHL (Cumulative Index to Nursing and Allied Health Literature), PsycINFO, Web of Science (WoS) and the Cochrane Library on the 17th April 2014. Screening, data extraction and assessment of methodological quality were done in duplicate. Our findings were used to develop a conceptual framework for transfer.

**Results:**

Our search identified 135 potential systematic reviews of which nine were included, reporting on 37 source studies, conducted in 19 different countries. Seven of nine (77.7%) of these reviews were high quality. There was some evidence for text messaging as a tool to improve adherence to antiretroviral therapy. Text messages also improved attendance at appointments and behaviour change outcomes. The findings were inconclusive for self-management of illness, treatment of tuberculosis and communicating results of medical investigations. The geographical distribution of text messaging research was limited to specific regions of the world. Prominent knowledge gaps included the absence of data on long term outcomes, patient satisfaction, and economic evaluations. The included reviews also identified methodological limitations in many of the primary studies.

**Conclusions:**

Global evidence supports the use of text messaging as a tool to improve adherence to medication and attendance at scheduled appointments. Given the similarities between HIV and other chronic diseases (long-term medications, life-long care, strong link to behaviour and the need for home-based support) evidence from HIV may be transferred to these diseases using our proposed framework by integration of HIV and chronic disease services or direct transfer.

## Background

HIV (human immune deficiency virus) is a major cause of morbidity and mortality all over the world. There are close to 2.6 million new infections each year, representing a 33 percent decline from 3.4 million, over the past ten years [1]. There has also been a drop in the number of AIDS (Acquired Immune Deficiency Syndrome) related deaths, due largely to the development and use of life-saving antiretroviral therapy (ART). As a result, there are more people living with HIV—over 35 million—many of whom are eligible for ART. However, treatment coverage is low worldwide, and among the people receiving ART, adherence to medication is low [1].

Mobile telephone technology is emerging as a tool in chronic disease management [2]. Mobile phone ownership and use is experiencing its greatest growth in Africa, where HIV is rife [3]. These two factors have led to the recent rise in research efforts regarding the use of mobile phones to enhance HIV care. On the African continent recent reports suggest that mobile phone text messages can be used to improve outcomes in people living with HIV [4-7].

Alongside HIV, tuberculosis and malaria benefit from considerable resources from The Global Fund (http://www.theglobalfund.org/en/about/diseases/). It is indisputable that these disease are responsible for many deaths in sub-Saharan Africa, yet, many other chronic non-communicable diseases (NCDs) still cause significant mortality and morbidity, owing in part to their long lasting nature and debilitating consequences. Close to 45% of the disease burden in adults living in low and middle-income countries can be attributed to NCDs [8]. In sub-Saharan Africa these chronic diseases are on the rise and do not receive sufficient attention [9]. Projections of current trends suggest that Africa will experience the highest increase in mortality (27% increase) from chronic conditions such as cardiovascular diseases, cancer and diabetes. Over the next ten years, 137 million people will die from a chronic disease [8]. There is also an unmet need for affordable medication for many NCDs in low and middle income countries [10], a problem resolved in HIV care through sustained commitments to disease control [10]. Addressing research, practice, and policy limitations are important steps towards alleviating the burden of disease due to chronic disease, however, many lessons can be learnt from the better resourced diseases like HIV, in which global investments have stabilized the epidemic by reducing the number of new cases and deaths [11]. Furthermore, people living with HIV are at a higher risk for developing many NCDs due to the effect of the virus itself (vascular damage), the effects of antiretroviral therapy (ART; which increases cardiovascular risk) and due to aging (because they are now living longer lives) [12]. The meeting point of HIV and NCDs present challenges for health systems in meeting up with the needs of the population, but also provides opportunities for transfer of knowledge. There is also a case to be made for extending HIV research to other chronic diseases as questions of equity arise when one considers the disproportionate distribution of funds in favor of HIV.

One critical similarity between HIV and the NCDs is that they both require long term and life-long medication. For this reason there is an enormous potential for poor adherence to medication and care. Secondly, both affect a considerably large portion of the population and thus merit concern as a public health problem [13]. The third factor is that they all have a strong behavioural component regarding their prevention, hence in both cases, behavioural modifications are required to reduce the risk of further complications. People living with HIV are encouraged to adopt healthy sexual practices to prevent the transmission of infection as well as potential acquisition of new strains from others; to take medication to control the disease; and to undergo frequent testing to monitor disease control. People with diabetes and high blood pressure, for example, are encouraged to modify their diets and incorporate exercise in their lifestyles, in addition to medication and testing. These behaviour modification similarities make it possible to apply information from HIV research to address certain issues in NCDs [12].

Both conditions also require considerable amounts of social support to sustain effective home-based-care. Family members may be involved in preparing meals that follow specific dietary restrictions, accompanying patients to the hospital, or administering medication.

With regards to health system weaknesses, HIV and NCDs share a number of barriers and challenges. These include demand-side barriers, inequitable availability of services, human resource shortages, limited adherence support, lack of infrastructure and equipment, unreliable drug and diagnostics supplies, poor referral and linkage systems, the need for community engagement; as well as stigma and discrimination [14].

HIV and NCDs have very different pathogeneses and clinical features [12]. The levels of stigma and discrimination with regards to HIV are considerably higher than for NCDs, where in some places NCDs are considered to be diseases of the rich in low-resource settings and people are therefore more likely to discuss such conditions more freely that they would with HIV. However for some NCDs such as diabetes, cancer, and mental disorders, there is still a considerable stigma [14]. The demographics are also quite different, with HIV affecting younger populations than the NCDs. Furthermore, in sub-Saharan Africa, women are disproportionately affected by HIV [13].

The purpose of the this paper is to map the scope of mobile phone text messaging research to identify opportunities for knowledge transfer, geographical coverage of text messaging research, and avenues for further research and scale-up. We describe the current state of the evidence, identify knowledge gaps, and highlight the points where lessons can be shared or more research is needed. We hope to use the available evidence on the application of text messaging in HIV and other chronic diseases to develop a framework for extension where the most appropriate kinds of interventions can be tested or taken to scale and duplication of efforts avoided.

### Why it is important to do this overview

In recent years, text messaging has emerged as an important communication tool in healthcare, yet there is limited information of what interventions work best and which should be taken to scale. In addition, given the disproportionate funding mechanisms for research, especially in low-resource settings, research efforts are directed toward conditions such as HIV, malaria and tuberculosis, often neglecting other chronic or NCDs. We purport that there is room for research findings to be transferred from the more favored conditions, given their similarities. Our objectives were to appraise the scope of text messaging interventions in health care and to develop a framework for transfer of research findings from HIV to NCDs.

## Methods

This paper is as systematic review of systematic reviews.

### Criteria for considering systematic reviews for inclusion

Our inclusion criteria were: full systematic reviews (with predetermined objectives, eligibility criteria, at least two data bases searched, data extraction, and quality assessment of included studies) that included at least one randomized trial, which examined the effectiveness of a text messaging intervention (automated or manual, two-way or one-way, irrespective of content) compared to no intervention or any other intervention, to improve a health or health-related outcome. The participants could be health workers (professional or lay persons) or consumers of health care (prevention or management). We excluded abstracts, non-systematic reviews and other overviews. We also excluded studies that addressed the broader field of mHealth, which would include smart phone applications (of which some may include text messages) and other portable medical devices.

### Search methods for identification of systematic reviews

We conducted an overview of systematic reviews to April 17, 2014 in the following electronic data bases and in all languages: PubMed, EMBASE (Exerpta Medica Database), CINAHL (Cumulative Index to Nursing and Allied Health Literature), PsycINFO, Web of Science (WoS) and the Cochrane Library. We sought systematic reviews of text messaging interventions in preventive or curative health care. We applied the following search terms in various combinations adapted for each database:

1. mobile phone OR cell* phone
2. text messag* OR SMS OR text*
3. *systematic review OR meta-analysis*

We also searched the reference lists of identified reviews and institutional websites including the World Health Organisation (WHO), the National Institute for Health and Care Excellence (NICE), and the Joint United Nations Programme on HIV/AIDS (UNAIDS).

### Systematic review selection, data collection and analysis

Duplicate citations were deleted, and the remaining abstracts were screened for relevance to our research questions. The abstracts of the retrieved citations were screened in duplicate by two authors (LM and SM). Full text manuscripts for relevant citations were retrieved and assessed for inclusion. We extracted data from included studies, using a piloted data extraction form, on the following: number and type of included studies, number of participants, target population, location of the studies, key conclusions, knowledge gaps and reporting quality. Data were extracted in duplicate (SM, LL) and verified by an adjudicator (LM). We assessed the methodological quality of the included systematic reviews using the AMSTAR (Assessment the methodological quality of systematic reviews) tool [15]. AMSTAR can be used to assess how well systematic reviews avoid bias against 11 criteria. Based on the number of items adequately reported a systematic review can be scored as high quality (8 to 11 items), medium quality (4 to 7 items) and low quality (3 or less items) [16]. The reviews were checked for overlap, since one study may be included in more than one systematic review. Agreement on screening and quality assessment was measured using the Kappa statistic, which measures agreement beyond chance [17]. Our findings are reported on the systematic review and source study level.

## Results

### Results of search

**Figure 1:**
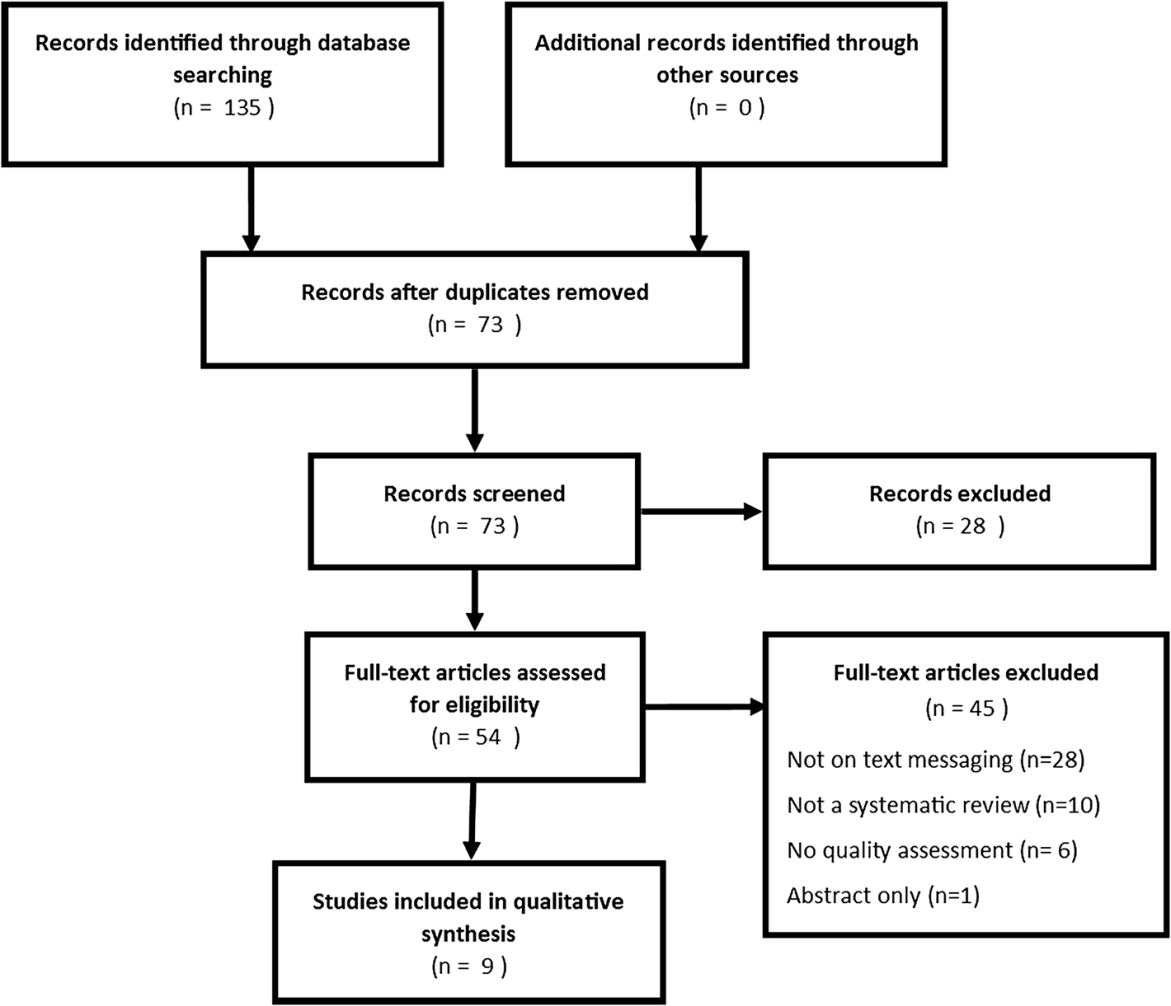
**PRISMA flow diagram of study selection.**

Our search retrieved 135 citations of which 74 abstracts were screened after removal of duplicates. Fifty-six (56) full text of relevant articles were retrieved and screened for eligibility by LL and SM. Agreement on screening of titles and abstracts was fair (Kappa = 0.34; 95% CI 0.12-0.55; p = 0.002). Citations for which there was disagreement were moved forward into the next round of screening. Agreement on screening of full text was high (Kappa = 0.94; 95% CI 0.81-1.00; p < 0.001). We included a total of 9 systematic reviews. The screening process is detailed in a PRISMA (Preferred Reporting Items for Systematic Reviews and Meta-Analyses) diagram (Figure 1).

### Description of included systematic reviews

The nine (9) included systematic reviews were all published in the English language, between 2010 and 2014, and reported on data from a total of 37 individual studies [6,18-25]. Five where Cochrane Collaboration reviews [6,18,21,22,25]. Five addressed text messaging interventions in non-specific populations [18,19,21,22,25], two focused on people living with HIV [6,20], one was on patients will tuberculosis [24], and one was on children and adolescents [23]. Among those with non-specific disease targets (i.e. HIV or tuberculosis), text messaging was investigated in the following diseases and health conditions: hypertension, diabetes, asthma, smoking, vitamin C, weight loss, physical activity, diet, circumcision and antenatal care. They covered a wide range of study designs, such as parallel group RCTs, crossover RCTs, quasi- experimental designs (non-randomized comparisons and before-after studies), and observational studies. In all the reviews the text messaging interventions targeted patients, and were used for the prevention of disease, disease management, or both. None of the interventions targeted health workers. The wide variety of participants, interventions and outcomes precluded meta-analyses in four systematic reviews. Details on the included studies are reported in Table 1.

**Table 1:** **Characteristics of included systematic reviews**

**Study ID (Reference)**	**Number and type of included studies**		**Target population**	**Health problem addressed in review (disease)**	**Prevention or Management**	**Location of studies (number)**	**Conclusions**	**Effect sizes**	**Knowledge gaps identified**
**Cole-Lewis 2010** [19]	12 studies: RCTs (9), Crossover RCTs (2), Quasi-experimental trial (1)	2408	Not specified	Behaviour change (weight loss, physical activity, diabetes, asthma, adherence to vitamin C)	Both	Canada (1), Finland (1), New Zealand (2), USA (2), France (1), Korea (2), UK (1), Croatia (1), and Austria (1)	There are short term effects on behavioral or clinical outcome related to disease prevention and management. Text messaging is a useful tool for behavior change interventions.	NA	1. Methodological limitations in studies
									2. Text message characteristics and combinations should be explored
									3. Long term effects should be investigated
**De Jongh 2013** [18]	4 studies: RCTs (4)	182	Not specified	Self-management of illness (diabetes, hypertension, asthma)	Management	Scotland (1), Croatia (1), USA (1), Spain (1)	Text messaging may support the self-management of long term conditions but have few direct impacts on health outcomes	NA	1. Limited evidence of efficacy
									2. Long-term effectiveness unknown
									3. Risks and limitations and consumer satisfaction are unknown
**Finitsis 2014** [20]	8 studies: RCTs (8)	1785	People living with HIV	Adherence to medication (HIV)	Management	USA (4), Kenya (2), Brazil (1), Cameroon (1)	Researchers should consider the adoption of a less than daily frequency of messaging that is individually timed and tailored and designed to evoke a reply from the recipient.	Odds ratio for adherence =1.39; 95% CI = 1.18-1.64 (8 RCTs)	1. Comparisons of design and intervention characteristics to obtain optimal effect are needed.
**Gurol-Urganci 2012** [22]	1 study: RCT (1)	2785	Not specified	Communicating results of medical investigations for anxiety (Down’s syndrome prenatal screen)	Management	Taiwan (1)	Unable to draw reliable conclusions dues to low quality of evidence coming from only one study. Positive and negative results delivered by text message may have different effects on anxiety	Mean anxiety score = −2.48; 95% CI-8.79 to 3.84 (1 RCT)	1. Methodological limitations in studies
									2. Some outcomes of interest are: health-seeking behaviour, patients’ evaluation of the intervention, costs, economic benefits, and potential adverse effects.
**Gurol-Urganci 2013 **[21]	8 studies: RCTs (8)	6615	Not specified	Attendance at healthcare appointments (not specified)	Both	China (2), UK (2), Malaysia (2), Kenya (1), Australia (1)	Mobile phone text message reminders increase healthcare appointment attendance rates when compared to no reminders and postal reminders. The current findings are insufficient to inform policy decisions	Relative risk for attendance rate at appointment = 1.14; 95% CI 1.03 to 1.26 (7 RCTs)	1. Methodological limitations in studies
									2. Some outcomes of interest include: health effects, adverse effects and harms, user evaluation of the intervention and user perceptions of its safety.
**Horvath 2012** [6]	2 studies: RCTs (2)	969	People living with HIV	Adherence to medication (HIV)	Management	Kenya (2)	Weekly text messages are efficacious in improving adherence to ART in resource limited settings and may be efficacious in suppressing viral load.	Risk ratio for non-adherence at 48–52 weeks = 0.78; 95% CI 0.68 to 0.89 (2 RCTs)	1. Larger RCTs in adolescent populations, and in persons who care for children and infants with HIV.
									2. Trials in high and middle-income countries are needed.
									3. Data on acceptability, and culture-specific issues such as message-content and message-length are needed.
**Militello 2012** [23]	6 studies: RCTs (4), Crossover RCT (1), Quasi-experimental trial (1)	433	Pediatric and adolescent populations	Health promotion (diabetes, antirejection medication adherence, physical activity, diet and sedentary behaviour)	Both	USA (3), UK (2), New Zealand (1), Austria (1)	Text messaging should be considered as an add-on to clinic care to improve health behaviours	NA	1. Methodological limitations in studies
									2. Long term effects and dose response data are of interest
**Nglazi 2013** [24]	4 studies: RCT (1), Observational (3) studies	565	Patients with tuberculosis	Adherence to medication (tuberculosis)	Management	Argentina (1), Kenya (1), South Africa (2)	The evidence is inconclusive on text messaging to improve adherence to TB treatment, but there is some potential	Risk ratio for adherence = 1.49; 95% CI 0.90 to 2.42 (one RCT).	1. Outcome measures for TB cure, successful completion of TB treatment, and development of drug resistance should be standardized.
**Vodopivec-Jamsek 2012** [25]	4 studies: RCTs (4)	1933	Not specified	Preventive health care (antenatal care, smoking, physical activity, diet and sedentary behaviour, adherence to vitamin C)	Prevention	Canada (1), Thailand (1), New Zealand (1), USA (1)	Text messages have the potential to contribute to health behaviour change in the short term alongside other media of health prevention information. .	NA	1. Long term effects are unknown
									2. Data is needed on costs, and possible risks and harms
									3. More information is needed for scale-up

NA = Not applicable (no pooled estimates); N = Total number of participants.

### Characteristics of text messaging interventions

Of the 37 source studies, 35 (94.6%) used mobile phones to deliver text messages. Two studies used text-enabled pager devices. Nine-teen (51.3%) of them delivered interactive messages (with the possibility of response from receiver or the sender), 24 (64.8%) used personalised messages, and 13 (35.1%) used automated delivery. Personalised messages were adapted to suit patient needs by tailoring of timing (to match timing of dosing, set according to patient preference or before appointments) and content (reminders, medical advice, appointment details or feedback on behaviour change activities). Text messages where used to improve adherence to medication (13 studies), as reminders for medical appointments (9 studies), for self-management of disease (for example, information on warning signs in pregnancy, and reminders to measure blood glucose to blood pressure; 8 studies), for behaviour change (for example, diet, sedentary time and exercise; 6 studies), and to deliver test results (1 study). Table 2 is a detailed description of the characteristics of the text messaging interventions.

**Table 2:** **Characteristics of text messaging interventions**

**Study name**	**Timing**	**Interactivity**	**Content**	**Duration**	**Automated**	**Personalisation**	**Condition**
**Benhamou 2007**	1/week	Yes	Medical advice based blood glucose	12 months	Not reported	Yes	Adult diabetics
**Bridges 2005**	Daily	No	Not reported	Not reported	Yes	Not reported	Patients on TB medication
**Broomhead 2012**	Daily on opening of pill bottle	No	Signal for opened pill bottle	Not reported	NA	NA	Patients on TB medication
**Chen 2008**	Once; 72 hours before appointment	No	Participants name and appointment details	NA	Yes	Yes	Adults with scheduled appointments
**Cheng 2008**	Once	No	Results of screening test	NA	Not reported	Yes	Women who underwent screening for Down's syndrome during pregnancy
**Cho 2009**	Every other week	Yes	Medical advice based blood glucose	3 months	Not reported	Yes	Adult diabetics
**Cocosila 2009**	1-2/day for 2 weeks;0-2/day intermittently for two weeks	Yes	Reminder to take Vitamin C	1 month	Yes	No	Healthy adults
**Da Costa 2012**	3/week	No	Not reported	5 months	Not reported	No	Adults with HIV
**Fairhurst 2008**	Once; about 12 hours before appointment	No	Not reported	NA	No	Not reported	Adults with scheduled appointments
**Franklin 2006**	1/week and daily	No	Reminder of goals set, tips and information and reminders to reinforce goal	12 months	Yes	Yes	Diabetes
**Haapala 2009**	Set by participant	Yes	Messages to reduce daily food intake, increase physical activity and encourage weight recording	12 month	Yes	Yes	Overweight adults
**Hanauer 2009**	Set by participants	Yes	Reminders to check blood glucose, with feedback provided with each submission of blood glucose and every Sunday	3 months	Not reported	Yes	Diabetics aged 12-25
**Hardy 2011**	Daily, matched to time of medication dosing	Yes	Not reported	1.4 months	Not reported	Yes	Adults with HIV
**Iribarren 2012**	2/week; and daily for defaulters	Yes	Education messages, reminders/check in	2 months	Yes	Not reported	Adults taking TB medication
**Jareethum 2008**	2/week	No	Information and warnings relating to abnormal symptoms	From 28 weeks gestation till delivery (about 3 months)	Not reported	Yes	Healthy pregnant women
**Koury 2005**	Once	No	Not reported	NA	Yes	No	Adults at ear-nose-throat clinic
**Leong 2006**	Once; 24–48 hours before appointment	No	Participants’ name and appointment details	NA	Not reported	Yes	Participants from primary care clinics
**Lester 2010**	1/week	Yes	Not reported	12 months	Not reported	No	Adults with HIV
**Liew 2009**	Once; 24–48 hours before appointment	No	Not reported	NA	Not reported	Yes	Patients with chronic diseases
**Lin 2012**	Twice per day on days 1 and 4 before the appointment	Not reported	Appointment details and importance of timely management	12 months	Not reported	Yes	Parents of children with cataracts scheduled for surgery
**Marquez-Contreras 2004**	2/week	No	Information on hypertension, compliance promotion, good health and dietary habits	6 months	Yes	No	Ambulatory hypertensive adults
**Mbuagbaw 2012**	1/week	Yes	Not reported	6 months	Not reported	No	Adults with HIV
**Miloh 2009**	1/day	Yes	Reminder messages sent to patient or caregiver to administer medication	12 months	Not reported	Yes	Liver transplant patients
**Musser 2001**	1/day	Yes	Not reported	0.5 months	Not reported	No	Adults with HIV
**Newton 2009**	1/week	No	Motivational	3 months	Yes	Not reported	Diabetic adolescents
**Odeny 2012**	Daily	No	Post-operative instructions and request to attend appointment	0.25 months	Not reported	Yes	Males who had undergone circumcision
**Ostojic 2005**	1/day	Yes	Medical advice on therapy based on PEF results	4 months	Not reported	Yes	Asthmatic adults
**Owiti 2012**	Once, a day before clinic appointment	No	Not reported	NA	Not reported	Yes	Patients on TB medication
**Patrick 2009**	2-5/day	Yes	Behavioural and dietary strategies, goal setting and weight monitoring	4 months	Yes	Yes	Overweight adults
**Pop-Eleches 2011**	1/week and 1/day	No	Not reported	12 months	Not reported	No	Adults with HIV
**Rami 2006**	4/day	Yes	Support for glycaemic control	3 months	Yes	Yes	Diabetic adolescents
**Rodgers 2005**	5x/day for 6 weeks then 3x/week for 20 weeks	Yes	Advice, support and distraction delivered in non-formal language	6.5 months	Yes	Yes	Adult smokers
Safren 2003*	Matched to time of medication dosing	No	Not reported	2.8 months	Not reported	Yes	Adults with HIV
**Shapiro 2008**	2/day 1 for child and 1 parent	Yes	Feedback message tailored to information on physical activity, sweetened beverage consumption and TV time	2 months	Yes	Yes	Children aged 5-13
Simoni 2009*	Matched to time of medication dosing	Yes	Not reported	3 months	Not reported	Yes	Adults with HIV
**Taylor 2012**	Once; 2 days before or on the day of appointment	Yes	Appointment details	NA	Not reported	Yes	Patients in need of physical therapy
**Yoon 2008**	Set by participants but at least 1/week	Yes	Treatment adjustment based on blood glucose	12 months	Not reported	Yes	Diabetic adults

*Text message pager devices.

### Excluded systematic reviews

We excluded 45 articles that did not meet our inclusion criteria. A full list of excluded studies and the reasons for exclusion is reported Table 3.

**Table 3:** **List of excluded studies**

**Reason for exclusion**	**Reference**
Abstract only	1. Kobos P. How has mobile phone text messaging been studied in pregnancy-related research? Communicating Nursing Research. 2013;**46**:718–18.
No quality assessment	2. Buchholz SW, Wilbur J, Ingram D, et al. Physical activity text messaging interventions in adults: a systematic review. Worldviews on evidence-based nursing. 2013;**10**:163–73.
	3. Buhi ER, Trudnak TE, Martinasek MP, et al. Mobile phone-based behavioural interventions for health: A systematic review. Health Education Journal. 2013;**72**:564–83.
	4. Deglise C, Suggs LS, Odermatt P. SMS for disease control in developing countries: a systematic review of mobile health applications. Journal of Telemedicine and Telecare. 2012;**18**:273–81.
	5. Guy R, Hocking J, Wand H, et al. How effective are short message service reminders at increasing clinic attendance? A meta-analysis and systematic review. Health Services Research. 2012;**47**:614–32.
	6. Head KJ, Noar SM, Iannarino NT, et al. Efficacy of text messaging-based interventions for health promotion: A meta-analysis. Social Science & Medicine. 2013;**97**:41–48.
	7. Williams AD. Use of a text messaging program to promote adherence to daily physical activity guidelines: A review of the literature. Bariatric Nursing and Surgical Patient Care. 2012;**7**:13–16.
Not a systematic review	8. Jardim C. Mobile phone-based interventions for smoking cessation. Sao Paulo Medical Journal. 2010;**128**:106–07.
	9. Lunny C, Taylor D, Memetovic J, et al. Short message service (SMS) interventions for the prevention and treatment of sexually transmitted infections: a systematic review protocol. Systematic reviews. 2014;**3**:7.
	10. Mbuagbaw L, Thabane L, Ongolo-Zogo P, et al. Trends and determining factors associated with adherence to antiretroviral therapy (ART) in Cameroon: a systematic review and analysis of the CAMPS trial. AIDS Res Ther. 2012;**9**:37.
	11. Mbuagbaw L, van der Kop ML, Lester RT, et al. Mobile phone text messages for improving adherence to antiretroviral therapy (ART): an individual patient data meta-analysis of randomised trials. BMJ Open. 2013;**3**.
	12. Mbuagbaw L, van der Kop ML, Lester RT, et al. Mobile phone text messages for improving adherence to antiretroviral therapy (ART): a protocol for an individual patient data meta-analysis of randomised trials. BMJ open. 2013;**3**.
	13. Shi C. Mobile phone messaging for facilitating self-management of long-term illnesses. International Journal of Evidence-Based Healthcare (Wiley-Blackwell). 2013;**11**:344–45.
	14. Agyapong VIO, Farren CK, McLoughlin DM. Mobile Phone Text Message Interventions in Psychiatry - what are the possibilities? Current Psychiatry Reviews. 2011;**7**:50–56.
	15. Bastawrous A, Armstrong MJ. Mobile health use in low-and high-income countries: An overview of the peer-reviewed literature. Journal of the Royal Society of Medicine. 2013;**106**:130–42.
	16. Blake H. Text messaging interventions increase adherence to antiretroviral therapy and smoking cessation. Evidence-Based Medicine. 2014;**19**:35–36.
	17. DiBello KKB, K. L.; Abrenica, S. C.; Worral, P. S. The effectiveness of text messaging programs on adherence to treatment regimens among adults aged 18 to 45 years diagnosed with asthma: A systematic review protocol. JBI Database of Systematic Reviews and Implementation Reports. 2013;**11**:170–85.
Not on text messaging exclusively	18. Holtz B, Lauckner C. Diabetes management via mobile phones: a systematic review. Telemed J E Health. 2012;**18**:175–84.
	19. Johnston W, Lederhausen A, Duncan J. Mobile technology: A synopsis and comment on "mobile phone-based interventions for smoking cessation". Translational Behavioral Medicine. 2013;**3**:231–32.
	20. Krishna S, Boren SA. Diabetes self-management care via cell phone: a systematic review. Journal of diabetes science and technology. 2008;**2**:509–17.
	21. Krishna S, Boren SA, Balas EA. Healthcare via Cell Phones: A Systematic Review. Telemedicine Journal and E-Health. 2009;**15**:231–40.
	22. Lau PWC, Lau EY, Wong DP, et al. A Systematic Review of Information and Communication Technology-Based Interventions for Promoting Physical Activity Behavior Change in Children and Adolescents. Journal of Medical Internet Research. 2011;**13**.
	23. Liang X, Wang Q, Yang X, et al. Effect of mobile phone intervention for diabetes on glycaemic control: a meta-analysis. Diabetic Medicine. 2011;**28**:455–63.
	24. Moussa MMR. Review on health effects related to mobile phones. Part II: results and conclusions. The Journal of the Egyptian Public Health Association. 2011;**86**:79–89.
	25. O'Reilly GA, Spruijt-Metz D. Current mHealth Technologies for Physical Activity Assessment and Promotion. American Journal of Preventive Medicine. 2013;**45**:501–07.
	26. Pellowski JA, Kalichman SC. Recent advances (2011–2012) in technology-delivered interventions for people living with hiv. Current HIV/AIDS Reports. 2012;**9**:326–34.
	27. Stephens J, Allen J. Mobile Phone Interventions to Increase Physical Activity and Reduce Weight A Systematic Review. Journal of Cardiovascular Nursing. 2013;**28**:320–29.
	28. Velthoven MHMMTv, Brusamento S, Majeed A, et al. Scope and effectiveness of mobile phone messaging for HIV/AIDS care: A systematic review. Psychology, Health & Medicine. 2013;**18**:182–202.
	29. Vervloet M, Linn AJ, van Weert JC, et al. The effectiveness of interventions using electronic reminders to improve adherence to chronic medication: a systematic review of the literature. Journal of the American Medical Informatics Association : JAMIA. 2012;**19**:696–704.
	30. Whittaker R, McRobbie H, Bullen C, et al. Mobile phone-based interventions for smoking cessation. Cochrane Database of Systematic Reviews. 2012.
	31. Wu RC, Tran K, Lo V, et al. Effects of clinical communication interventions in hospitals: A systematic review of information and communication technology adoptions for improved communication between clinicians. International journal of medical informatics. 2012;**81**:723–32.
	32. Barnighausen TC, K.; Chimbindi, N.; Peoples, A.; Haberer, J.; Newell, M. L. Interventions to increase antiretroviral adherence in sub-Saharan Africa: A systematic review of evaluation studies. The Lancet infectious diseases. [Review]. 2011;**11**:942–51.
	33. Braun R, Catalani C, Wimbush J, et al. Community health workers and mobile technology: a systematic review of the literature. PloS one. 2013;**8**:e65772.
	34. Catalani C, Philbrick W, Fraser H, et al. mHealth for HIV treatment & prevention: A systematic review of the literature. Open AIDS Journal. 2013;**7**:17–41.
	35. Chavez NR, Shearer LS, Rosenthal SL. Use of digital media technology for primary prevention of STIS/HIV in adolescents and young adults: A systematic review of the literature. Journal of Adolescent Health. 2013;**52**:S84-S85.
	36. Chen YF, Madan J, Welton N, et al. Effectiveness and cost-effectiveness of computer and other electronic aids for smoking cessation: A systematic review and network meta-analysis. Health Technology Assessment. 2012;**16**:1–205.
	37. Connelly J, Kirk A, Masthoff J, et al. The use of technology to promote physical activity in Type 2 diabetes management: a systematic review. Diabetic Medicine. 2013;**30**:1420–32.
	38. Cotter AP, Durant N, Agne AA, et al. Internet interventions to support lifestyle modification for diabetes management: A systematic review of the evidence. Journal of diabetes and its complications. 2014;**28**:243–51.
	39. Fanning J, Mullen SP, McAuley E. Increasing Physical Activity With Mobile Devices: A Meta-Analysis. Journal of Medical Internet Research. 2012;**14**:159–69.
	40. Free C, Phillips G, Watson L, et al. The effectiveness of mobile-health technologies to improve health care service delivery processes: a systematic review and meta-analysis. PLoS medicine. 2013;**10**:e1001363.
	41. Gurman TAR, S. E.; Roess, A. A. Effectiveness of mHealth behavior change communication interventions in developing countries: a systematic review of the literature. Journal of health communication. [Review]. 2012;**17 Suppl 1**:82–104.
	42. Guse K, Levine D, Martins S, et al. Interventions Using New Digital Media to Improve Adolescent Sexual Health: A Systematic Review. Journal of Adolescent Health. 2012;**51**:535–43.
	43. Hasvold PE, Wootton R. Use of telephone and SMS reminders to improve attendance at hospital appointments: A systematic review. Journal of Telemedicine and Telecare. 2011;**17**:358–64.
	44. Haug S, Sannemann J, Meyer C, et al. Internet and Mobile Phone Interventions to Decrease Alcohol Consumption and to Support Smoking Cessation in Adolescents: A Review. Gesundheitswesen. 2012;**74**:160–77.
	45. Whittaker R, McRobbie H, Bullen C, et al. Mobile phone-based interventions for smoking cessation. Cochrane Database of Systematic Reviews. 2012.

### Quality of included systematic reviews

The median AMSTAR ranking was 9 (first quartile (Q1): 7.5; third quartile (Q3): 10). Two reviews (22.2%) were rated as medium quality [19,23] and seven (77.7%) were rated as high quality [6,18,20-25]. Agreement on the AMSTAR rankings was moderate (estimated Kappa = 0.53 95% CI 0.31-0.74; p < 0.001). The items for which there was a lot of disagreement were scoring whether publication bias was assessed and whether conflict of interest was included in the review. Disagreements were resolved by adjudication of a third author (LM) using AMSTAR documentation [15]. The final AMSTAR rankings reflect the guidance from this document. Most studies did not report on conflicts of interest. A full report of the AMSTAR rankings is reported in Table 4.

**Table 4:** **Methodological quality of included studies using the AMSTAR checklist**

**CRITERIA**	**Cole-Lewis 2010 [** 19 **]**	**De Jongh 2012 [** 18 **]**	**Finitsis 2014 [** 20 **]**	**Gurol-Urganci 2012 [** 22 **]**	**Gurol-Urganci 2013 [** 21 **]**	**Horvath 2012 [** 6 **]**	**Militello 2012 [** 23 **]**	**Nglazi 2013 [** 24 **]**	**Vodopivec-Jamsek 2012 [** 25 **]**
**Was an 'a priori' design provided?**	0	1	0	1	1	1	1	1	1
**Was there duplicate study selection and data extraction?**	0	1	1	1	1	1	1	1	1
**Was a comprehensive literature search performed?**	1	1	1	1	1	1	1	1	1
**Was the status of publication (i.e. grey literature) used as an inclusion criterion?**	0	1	1	1	1	1	0	1	1
**Was a list of studies (included and excluded) provided?**	0	1	1	1	1	1	0	1	1
**Were the characteristics of the included studies provided?**	1	1	1	1	1	1	1	1	1
**Was the scientific quality of the included studies assessed and documented?**	1	1	1	1	1	1	1	1	1
**Was the scientific quality of the included studies used appropriately in formulating conclusions?**	1	1	1	1	1	1	1	1	1
**Were the methods used to combine the findings of studies appropriate?**	1	1	1	1	1	1	1	1	1
**Was the likelihood of publication bias assessed?**	0	0	0	1	1	1	0	0	0
**Was the conflict of interest included?**	0	0	0	0	1	0	0	0	0
**AMSTAR scores**	5	9	8	10	10	10	7	9	9
**Ranking**	**Medium**	**High**	**High**	**High**	**High**	**High**	**Medium**	**High**	**High**

### Effects of text messaging interventions in HIV

The findings from two high quality systematic reviews suggest that text messaging can be used in HIV to improve adherence to medication, as well as biological outcomes such as viral load [6,20].

### Effects of text messaging interventions for other conditions

Only one high quality review investigated the effects of text messaging on adherence to tuberculosis medication, and the evidence was inconclusive [24]. Two medium quality reviews targeted health promotion in pediatric and adolescent populations, and behaviour change across many conditions. They both supported the use of text messaging to promote health behaviour change [19,23]. One high quality review described limited direct impact on health outcomes when text messaging was used for self-management of illness [18]. Two high quality reviews found that text messages increased attendance at scheduled appointments, and may be used in preventive health care alongside other interventions [21,25]. The findings on the use of text messages to communicate results of medical investigations, from one high quality review, were inconclusive [22].

### Locations of included studies

**Figure 2:**
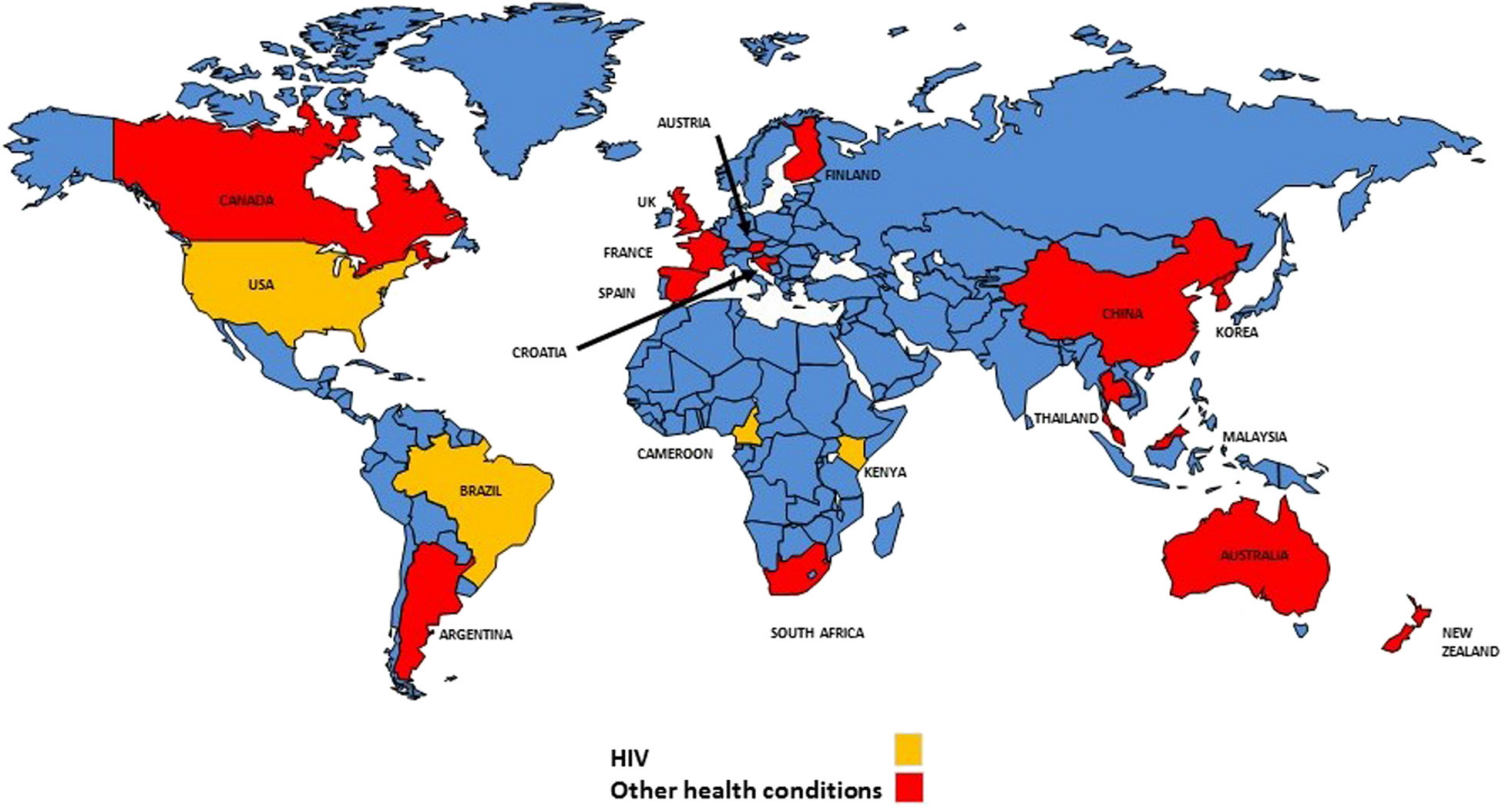
**World map illustrating geographical distribution of text messaging interventional research as of May 2014 (Map developed courtesy of PowerPoint Toolkit:** **http://ppt-toolkit.com/** **).**

These 37 studies were conducted in 19 countries. Eight (8) studies were conducted in the USA, four (4) in Kenya, three (3) the UK, two (2) each in Malaysia, China, Korea, South Africa, New Zealand and one (1) each in Argentina, Australia, Austria, Brazil, Cameroon, Canada, Croatia, Finland, France, Spain and Thailand. Figure 2 is a world map highlighting the locations where text messaging intervention research in this study were conducted.

### Knowledge gaps identified

These systematic reviews identified many knowledge gaps in the literature and avenues for further research. Most of them addressed the need for higher quality studies [19,21-23], as well as the investigation of long-term effects [18,19,23]. Three studies highlighted the importance of delving further into the characteristics of the text messages in order to optimise outcomes [6,19,20]. Other knowledge gaps identified included risks and harms [18,21,22,25], user satisfaction and acceptability [18,21,22], cost effectiveness [18,21,25], and dose response [23]. A full description of the knowledge gaps identified is reported in Table 1.

### Overlap of systematic reviews

The 9 included systematic reviews reported on a total of 49 studies, relating to 37 unique studies. Two studies appeared in three reviews, and 8 studies were included in two reviews. The overlap was highest among the systematic reviews with broad target populations and health problems addressed [18,19,23,25]. Both studies included in Horvath’s 2012 systematic review [6] were included in Finitsis et al’s 2014 systematic review [20]. This overlap, and all others were taken into account in the mapping process and source studies were considered only once.

## Discussion

### What is the state of the evidence of mobile phone text messaging technology for management and prevention of HIV and other diseases?

A considerable body of evidence is building in favour of text message interventions for improving health outcomes. Text message interventions are tested across a wide variety of conditions for prevention and management. There is room for the exploration of text messaging in younger populations, care givers, and in higher income settings. The only behaviour change intervention targeted in people living with HIV was adherence to medication. The effects on behaviour change and health promotion are inconsistent, and almost null for self- management of illness, communicating results of medical investigations and adherence to TB medication. Though the results seem promising, almost all the included systematic reviews highlighted important methodological limitations in the source studies, and the need to investigate the effects of text messaging on long term outcomes. Text messaging is used for a wide variety of purposes, medical conditions and in different formats. The commonality between all the text messaging interventions is enhanced communication between health care providers and consumers. Though challenging to evaluate, client-provider communication is a critical aspect of health, and would explain the strength of two-way messaging over one way messaging.

### What is the geographical coverage of text messaging interventions in the world?

The locations of the source studies were unevenly distributed around the globe. Eight studies on HIV were located in four countries: four in the US, two in Kenya, and one each in Cameroon and Brazil. There were no text messaging studies for NCDs conducted in Africa and only one on improving attendance at scheduled appointments, in the general population. There were no HIV studies in Europe and Asia.

### How can the application of the technology be transferred from HIV to NCDs?

Two approaches can be adopted in extending HIV research to NCDs. The first is integration of services [12]. If HIV services and NCD services were integrated, a lot could be gained in terms of shared human, financial, material and community resources. Yet, given the often separate funding mechanisms for these vertical programmes and persistent stigma and discrimination against people living with HIV, such integration may be less well received than integration of tuberculosis and HIV services or antenatal care with prevention of mother to child transmission of HIV – both of which are more similar in terms of service provision. However, it is important to consider that modern HIV management will likely result in the development of concurrent chronic diseases in patients. Some programs have successfully integrated HIV care with NCD in Cambodia and Kenya [26]. The second approach would be transferring evidence-based practices or leveraging HIV successes for NCD care. Successes in HIV which contributed to controlling the epidemic include: improved planning and managing of resources, better financing mechanisms, human resources strengthening, augmented infrastructure, developing and strengthening supply systems, better data management and clinical services and behaviour change interventions among patients and providers [14]. Such transfers may be easier to implement if the evidence developed from HIV research is applicable to NCDs.

In the field of health economics, a number of guidelines have been published to determine if an intervention is suitable to be transferred from one geographic location to another. Some of these can also be used, albeit with some modification, to determine if technologies are transferable across disease conditions. Borrowing from Heyland’s Generalizability criteria, items such as: similarities between patients, clinical setting, cost, outcome measures and safety must be considered [27].

Despite the difference in demographics between people living with HIV and those with NCDs, we now recognise the accrued risk of NCDs, especially cardiovascular diseases in people with HIV [28]. Owing to the fact the people living with HIV are now living longer lives, they are more likely to merge with the age group of people who experience NCD morbidity and mortality. The demographics are becoming more and more similar. With regards to text messaging only level of education seems to be a factor affecting response [29].

The cost of care for HIV is considerably higher than for NCDs, but many of these costs are reduced in places where testing and ART are provided free of charge. However, there is no reason to suspect that a text messaging intervention will cost more for NCDs compared to HIV.

Similar outcomes are of interest in both conditions, notably adherence to medication and lifestyle modifications, both of which can be achieved using mobile phone technology [30,31].

In addition to these generalizability criteria, the flexibility of text messaging interventions as seen in the diversity of intervention types is an advantage for transfer. Personalisation, timing and content can be modified to adapt a text messaging intervention to another population or health system. Automated messages, may reduce the human resource requirements but limit interactivity. However, many of these interventions were used in an experimental setting on a small number of participants, and it is unclear how they will perform on a large scale.

The more challenging question relates to geographical transfer. Given the marked difference in health systems, disease burden, culture and use of mobile phones, the most appropriate approach to transfer may involve efforts to replicate or improve research findings in other settings.

### Framework

**Figure 3:**
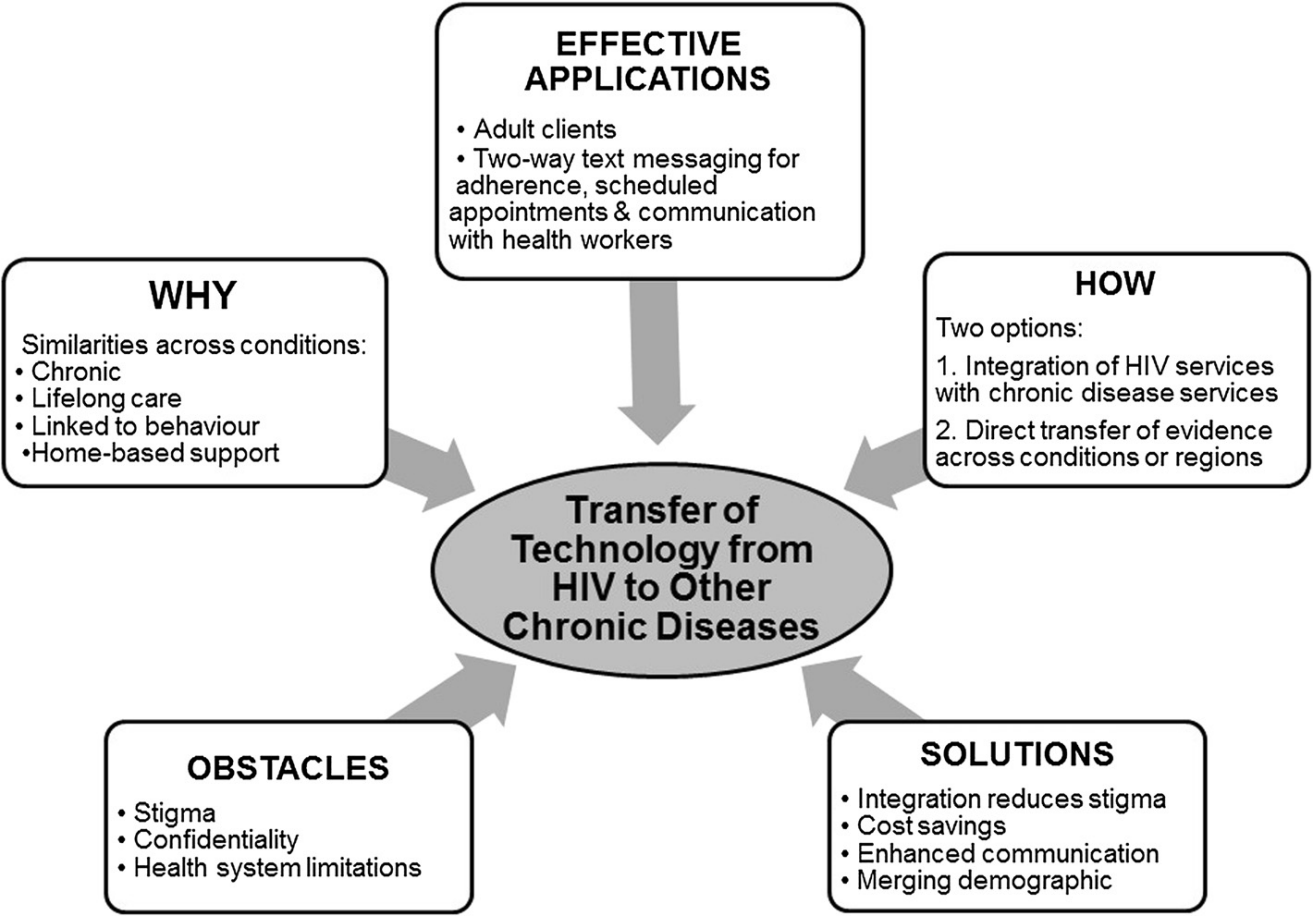
**A framework for transfer of text messaging evidence in HIV and chronic diseases.**

Building upon the existing body of evidence, we propose a conceptual framework for the transfer and implementation of research findings from HIV to NCDs. This framework highlights why it is appropriate to transfer text messaging technology from HIV to NCDs (they are both chronic conditions requiring lifelong care, are strongly linked to behaviour, and often require home-based support). We propose two options for integration: the integration of HIV services with NCD services (providing care for NCDs alongside HIV care with the same staff in the same clinics so that text messaging services can be provided for both conditions) or direct transfer of evidence (a replication of text messaging HIV services in NCD clinics within the same facility of another). Current evidence supports two-way text messaging interventions for adult clients to improve adherence to medication, attendance at scheduled appointments and communication with health workers. The obstacles to transfer we identified are stigma, which can be addressed through education and integration of services; issues related to confidentiality, which can be addressed using appropriate content or encryption in the text messages; and health system limitations such as lack of infrastructure and human capital. The transfer process can be facilitated by integration of services, potential saved costs, enhanced client-provider communication and the fact that the HIV and NCD demographics are merging – services will be provided to an overlapping group of clients. The items for which further research is required should also be considered, and include the use of text messaging services in caregivers and adolescents, longer term effects (on clinical viral load, acceptability and user fatigue) and some NCD specific outcomes such as blood pressure control, blood sugar control, weight control and cholesterol control. This framework is summarised in Figure 3.

### Limitations and strengths

This study is not without limitations, notably the stringent inclusion criteria for systematic reviews, which may have led to the exclusion of some text messaging studies that were considered in other reviews under the canopy of mHealth. Some text messaging studies that did not make it into reviews, either because they did not meet individual systematic review inclusion criteria or are more recent would have been missed in this overview. In addition, many studies were unable to produce a pooled estimate of effects due to diversity in study design, participants and outcomes. The strengths of this study include a geographic mapping, a summary of knowledge gaps, and a framework for transfer of evidence generated for HIV to other chronic conditions.

## Conclusions

### Implications for practice

Individual medical practices can use text messaging interventions to improve adherence to medication and attendance at clinical appointments, as long as the correct precautions relating to patient confidentiality are incorporated.

### Implications for research and policy

A systematic survey of the evidence demonstrates a paucity of research evidence on how text messages can be used to improve care in patients with NCDs in Africa. The findings from HIV research can for the most part be transferred to NCDs and may represent cost savings for donors, governments and taxpayers. Given that the conditions are not identical, we recommend a gradual approach to technology transfer, building on experiences from other geographical regions. Interestingly, despite high rates of TB/HIV, comorbidity there isn’t much evidence supporting the use of text messaging to enhance TB outcomes. This would be a worthwhile and conservative step towards extending evidence from HIV research.

Text messaging research should be conducted with adolescent populations with HIV, and people who care for children or adults with HIV. Other populations of interest for text messaging research include health care professionals, and community health workers. The current evidence is in favor of weekly, two-way messaging, but “dose response”, the possibility of user fatigue and in-depth understanding of messaging characteristics warrant further investigation. Given that text messaging is emerging as an add-on to usual care, and may also support other adherence enhancing interventions, comparisons to other interventions may mask its value, yet comparisons of different contents, durations and combinations would be informative. We recommend that further research focus on long term clinical outcomes like viral load and exploration of any harms that may occur (notably inadvertent disclosure of status or road traffic accidents related to the use of text messaging). In addition, strategies to ensure confidentiality should be developed. Finally, economic evaluations of the cost effectiveness for text messaging interventions would be critical components of the big picture.

## References

[1] Global Report: UNAIDS report on the global AIDS epidemic 2013. [http://www.unaids.org/sites/default/files/en/media/unaids/contentassets/documents/epidemiology/2013/]

[2] Lester RT, Karanja S (2008). Mobile phones: exceptional tools for HIV/AIDS, health, and crisis management. Lancet Infect Dis.

[3] Lester RT, Gelmon L, Plummer FA (2006). Cell phones: tightening the communication gap in resource-limited antiretroviral programmes?. AIDS.

[4] Lester RT, Ritvo P, Mills EJ, Kariri A, Karanja S, Chung MH (2010). Effects of a mobile phone short message service on antiretroviral treatment adherence in Kenya (WelTel Kenya1): a randomised trial. Lancet.

[5] Pop-Eleches C, Thirumurthy H, Habyarimana JP, Zivin JG, Goldstein MP, de Walque D (2011). Mobile phone technologies improve adherence to antiretroviral treatment in a resource-limited setting: a randomized controlled trial of text message reminders. AIDS.

[6] Horvath T, Azman H, Kennedy GE, Rutherford GW (2012). Mobile phone text messaging for promoting adherence to antiretroviral therapy in patients with HIV infection. Cochrane Database Syst Rev.

[7] Mukund Bahadur KC, Murray PJ (2010). Cell phone short messaging service (SMS) for HIV/AIDS in South Africa: a literature review. Stud Health Technol Inf.

[8] Preventing Chronic Disease. A vital investment. [http://www.who.int/healthinfo/global_burden_disease/2004_report_update/en/]

[9] de-Graft Aikins A, Unwin N, Agyemang C, Allotey P, Campbell C, Arhinful D (2010). Tackling Africa’s chronic disease burden: from the local to the global. Global Health.

[10] Bollyky TJ (2013). Access to drugs for treatment of noncommunicable diseases. PLoS Med.

[11] Report on the Global AIDS Epidemic. [http://www.unaids.org/globalreport/Global_report.htm]

[12] Nigatu T (2012). Integration of HIV and noncommunicable diseases in health care delivery in low- and middle-income countries. Prev Chronic Dis.

[13] World Health Statistics. [http://apps.who.int/iris/bitstream/10665/44844/1/9789241564441_eng.pdf?ua=1]

[14] Rabkin M, El-Sadr WM (2011). Why reinvent the wheel? Leveraging the lessons of HIV scale-up to confront non-communicable diseases. Glob Public Health.

[15] Shea BJ, Hamel C, Wells GA, Bouter LM, Kristjansson E, Grimshaw J (2009). AMSTAR is a reliable and valid measurement tool to assess the methodological quality of systematic reviews. J Clin Epidemiol.

[16] Shea BJ, Grimshaw JM, Wells GA, Boers M, Andersson N, Hamel C (2007). Development of AMSTAR: a measurement tool to assess the methodological quality of systematic reviews. BMC Med Res Methodol.

[17] Viera AJ, Garrett JM (2005). Understanding interobserver agreement: the kappa statistic. Fam Med.

[18] de Jongh T, Gurol-Urganci I, Vodopivec-Jamsek V, Car J, Atun R (2012). Mobile phone messaging for facilitating self-management of long-term illnesses. Cochrane Database Syst Rev.

[19] Cole-Lewis H, Kershaw T (2010). Text messaging as a tool for behavior change in disease prevention and management. Epidemiol Rev.

[20] Finitsis DJ, Pellowski JA, Johnson BT. Text message intervention designs to promote adherence to antiretroviral therapy (ART): A meta-analysis of randomized controlled trials. PLoS ONE 2014;9:e88166. 10.1371/journal.pone.0088166 PMC3914915 24505411

[21] Gurol-Urganci I, de Jongh T, Vodopivec-Jamsek V, Atun R, Car J (2013). Mobile phone messaging reminders for attendance at healthcare appointments. Cochrane Database Syst Rev.

[22] Gurol-Urganci I, de Jongh T, Vodopivec-Jamsek V, Car J, Atun R (2012). Mobile phone messaging for communicating results of medical investigations. Cochrane Database Syst Rev.

[23] Militello LK, Kelly SA, Melnyk BM (2012). Systematic review of text-messaging interventions to promote healthy behaviors in pediatric and adolescent populations: implications for clinical practice and research. Worldviews Evid Based Nurs.

[24] Nglazi MD, Bekker LG, Wood R, Hussey GD, Wiysonge CS (2013). Mobile phone text messaging for promoting adherence to anti-tuberculosis treatment: a systematic review. BMC Infect Dis.

[25] Vodopivec-Jamsek V, de Jongh T, Gurol-Urganci I, Atun R, Car J (2012). Mobile phone messaging for preventive health care. Cochrane Database Syst Rev.

[26] Janssens B, Van Damme W, Raleigh B, Gupta J, Khem S, Soy Ty K (2007). Offering integrated care for HIV/AIDS, diabetes and hypertension within chronic disease clinics in Cambodia. Bull World Health Organ.

[27] Heyland DK, Kernerman P, Gafni A, Cook DJ (1996). Economic evaluations in the critical care literature: do they help us improve the efficiency of our unit?. Crit Care Med.

[28] Hemkens LG, Bucher HC (2014). HIV infection and cardiovascular disease. Eur Heart J.

[29] Mbuagbaw L, van der Kop ML, Lester RT, Thirumurthy H, Pop-Eleches C, Ye C, Smieja M, Dolovich L, Mills EJ, Thabane L. Mobile phone text messages for improving adherence to antiretroviral therapy (ART): an individual patient data meta-analysis of randomised trials. BMJ Open 2013;3:e002954. 10.1136/bmjopen-2013-003950 PMC3884740 24345901

[30] Wizner B, Gaciong Z, Narkiewicz K, Grodzicki T (2010). High risk patients benefit most from the Sms-based intervention: Pp.20.272. J Hypertens.

[31] Ramachandran A, Snehalatha C, Ram J, Selvam S, Simon M, Nanditha A (2013). Effectiveness of mobile phone messaging in prevention of type 2 diabetes by lifestyle modification in men in India: a prospective, parallel-group, randomised controlled trial. Lancet Diabetes Endocrinol.

